# *Curvularia spicifera* in Non-Invasive Fungal Rhinosinusitis: Case Reports and Diagnostic Insights

**DOI:** 10.3390/pathogens15050523

**Published:** 2026-05-13

**Authors:** Nicola Ferraro, Elizabeth Iskandar, Antonino Maria Guglielmo Pitrolo, Marina Ramus, Fabio Pagella, Sveva Introini, Fausto Baldanti, Caterina Cavanna

**Affiliations:** 1 Microbiology and Virology Department, Fondazione I.R.C.C.S. Policlinico San Matteo, 27100 Pavia, Italy; n.ferraro@smatteo.pv.it (N.F.); e.iskandar@smatteo.pv.it (E.I.);; 2 Regional Centre for Infectious Diseases (CEREMI), Lombardy Region, Italy; 3 National PhD Programme in One Health Approaches to Infectious Diseases and Life Science Research, Department of Public Health, Experimental and Forensic Medicine, University of Pavia, 27100 Pavia, Italy; 4 Department of Otorhinolaryngology, Fondazione I.R.C.C.S. Policlinico San Matteo, 27100 Pavia, Italysveva.introini01@universitadipavia.it (S.I.); 5 Department of Clinical, Surgical, Diagnostic and Pediatric Sciences, University of Pavia, 27100 Pavia, Italy

**Keywords:** *Curvularia spicifera*, fungus ball, allergic mycotic rhinosinusitis, immunocompetent patients, cortisone

## Abstract

The clinical cases described in this text add to the limited literature on chronic and allergic rhinosinusitis caused by dematiaceous fungi, particularly *Curvularia spicifera*. These cases highlight the growing recognition of fungal infections as a significant factor in the etiology of rhinosinusitis, a condition traditionally attributed to bacterial causes It has become evident that a comprehensive clinical approach, involving diagnostic imaging and laboratory examinations, particularly culture-based analysis, has been crucial in identifying the specific fungal pathogen responsible for the infection. Additionally, molecular biology techniques have proven indispensable in enhancing diagnostic accuracy and the understanding of such infections. Importantly, these types of infections are commonly observed in immunocompetent individuals, distinguishing them from other fungal infections that typically affect immunocompromised patients. This study underlines the importance of integrating microbiological findings with clinical, radiological, and histopathological data for the accurate diagnosis of non-invasive fungal rhinosinusitis, particularly given the lack of serological assays specific for this species. The available literature on these infections remains limited, and diagnosis continues to rely on an integrated multimodal approach.

## 1. Introduction

Fungal rhinosinusitis is categorized into two main forms: invasive and non-invasive. Among the causes of the non-invasive form, dematiaceous fungi are among the most common pathogens, although *Aspergillus fumigatus* is the most common etiological agent [1,2]. Non-invasive fungal rhinosinusitis is further categorized into fungus ball, allergic fungal rhinosinusitis (AFRS), and eosinophilic mucin chronic rhinosinusitis. Allergic fungal rhinosinusitis is estimated to account for 6–9% of rhinosinusitis cases that require surgical intervention [3]. *Curvularia spicifera* is a dematiaceous fungus, often cited in the literature under outdated synonyms, which can lead to confusion. Historically used names include *Bipolaris spicifera*, *Brachycladium spiciferum*, *Drechslera spicifera*, and *Helminthosporium spiciferum*. Additionally, *Cochliobolus spiciferus* represents the teleomorphic (sexual) stage of this species [4]. In this work, we describe two cases of non-invasive fungal rhinosinusitis caused by *Curvularia spicifera*, both managed surgically with favourable outcomes. In addition, we performed a review of the literature to contextualize our findings, focusing on previously reported cases of *C. spicifera* rhinosinusitis, their clinical manifestations, diagnostic approaches, and management strategies.

## 2. Case Presentation

### 2.1. Case 1

**Figure 1:**
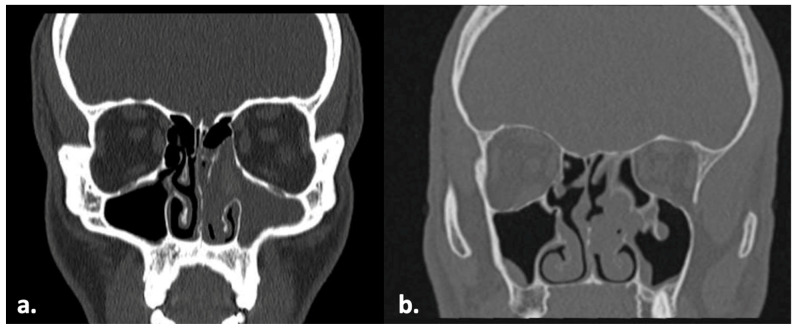
A CT scan of the paranasal sinuses showing the affected maxillary and ethmoid sinus opacification of patient 1 (**a**) and patient 2 (**b**) with areas of increased attenuation.

A 40-year-old male patient with a past medical history of bronchial asthma presented with nasal obstruction, associated anterior rhinorrhea, and hyposmia. The patient denied headache, facial pain, recurrent epistaxis, and cacosmia. He was on topical nasal therapy for allergic rhinitis and had undergone functional endoscopic sinus surgery (FESS) in October 2020. The most recent assumption of systemic corticosteroids was in January 2024. A maxillofacial CT scan without contrast enhancement of the paranasal sinuses performed in December 2023 demonstrated opacification of the paranasal sinuses, predominantly involving the anterior sinonasal compartments, associated with diffuse mucosal thickening and polypoid mucosal hypertrophy (Figure 1a). A septoplasty was also performed, followed by a revision endoscopic sinus surgery, during which biopsy samples of the polypoid neoformation were obtained and showed sand concretions suggestive of fungal origin in the maxillary, ethmoidal, sphenoidal, and frontal sinuses. Muco-purulent secretions were also drained intraoperatively and sent to the Microbiology and Virology Laboratory for culture examination. Additionally, biopsy specimens of the paranasal sinus mucosa were collected and submitted to the Pathology Department for histological evaluation. A histological examination of the polypoid specimens revealed an inflammatory polyp with a conspicuous eosinophilic infiltrate. In addition, areas of necrotic and inflammatory debris containing very few fungal hyphae and spores were identified in several fragments. The respiratory mucosa biopsies showed edema and chronic inflammatory changes. The culture of the material collected from the right and left frontal sinuses and the left nasal cavity yielded fungal growth. The species identification of *Curvularia spicifera* was subsequently confirmed by ITS region sequencing (see Section 3). At the postoperative follow-up in August 2025, the patient reported good general health, with no specific sinonasal symptoms.

### 2.2. Case 2

A 28-year-old male patient reported a one-year history of bilateral nasal obstruction, more pronounced on the left side, associated with anterior rhinorrhea. He also complained of obligatory oral breathing both day and night, hyposmia, cacosmia and dysgeusia. The patient denied facial pain and frontal headache. His past medical history included bronchial asthma of allergic etiology, managed with ongoing therapy. He was treated with topical corticosteroids with only partial clinical improvement. A maxillofacial CT scan of the paranasal sinuses performed in June 2024 without contrast enhancement demonstrated extensive opacification of the left paranasal sinuses involving multiple sinus cavities, consistent with chronic pansinusitis (Figure 1b). The most recent otorhinolaryngologic evaluation in late August 2024 showed chronic rhinosinusitis with acute exacerbation on nasal endoscopy, leading to the indication for elective endoscopic sinus surgery (ESS) based on clinical and radiologic findings. The nasal endoscopic evaluation showed polypoid neoformations partially obstructing the left nasal cavity with allergic mucin. Biopsies of the polypoid lesions were also collected and submitted for histopathological analysis. The muco-purulent material was drained from the sinuses. All the paranasal sinuses were opened and found to be filled with allergic mucin, which was removed and sent for histological and microbiological examination. A histologic examination of the polypoid fragments revealed inflammatory nasal polyps with a moderate eosinophilic infiltrate, focal epithelial erosion, and areas of squamous metaplasia. The mucin contained scattered inflammatory cells and a moderate number of eosinophilic granulocytes. The culture of the material obtained from the maxillary and frontal sinuses yielded fungal growth. The molecular ITS sequencing of the grown fungus confirmed the species identification of *Curvularia spicifera* (see Section 3).

Overall, the clinical, radiological, histopathological, and microbiological findings of both cases were consistent with non-invasive fungal rhinosinusitis caused by *Curvularia spicifera*, successfully managed with endoscopic sinus surgery and short-term corticosteroid therapy, without the use of antifungal treatment.

In both cases, histopathological examination revealed the presence of a conspicuous component of eosinophilic infiltrates, attributable to the inflammatory state, while the presence of a few fungal hyphae and spores was observed microscopically in a polypoid fragment in one case. Additionally, eosinophil-rich mucin was observed in the sinus cavities, accompanied by characteristic radiographic findings [5].

## 3. Materials and Methods

**Figure 2:**
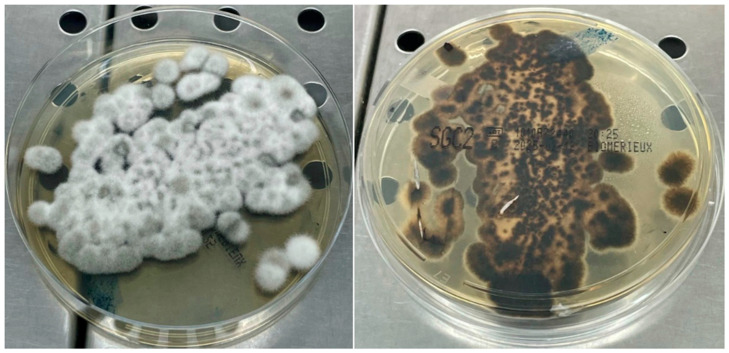
Rapidly growing colonies that transition from white or greyish to dark brown or black with a velvety texture, colonies grown (day 6) on Sabouraud Dextrose Agar (SDA), both the colony surface (**left**) and reverse (**right**).

**Figure 3:**
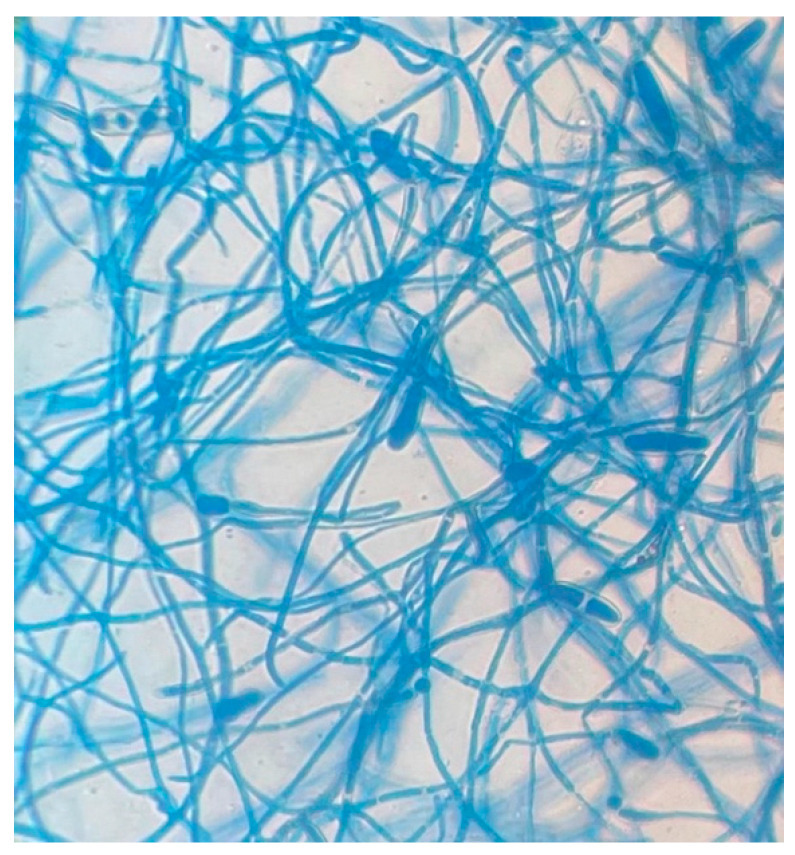
Microscopy characterization (40×) of *C. spicifera* directly from grown culture on day 5 using lactophenol cotton blue stain; it has curved, multi-celled conidia with swollen central cell, giving it boat-like appearance.

In both cases, the collected materials were cultured on Sabouraud Dextrose Agar (SDA) and incubated at a temperature of both 37 °C and 30 °C to promote optimal fungal growth, facilitating the isolation of potential pathogens. After a culture period of six days, colonies exhibiting moderate growth patterns were observed, characterized by a downy consistency and an initial greyish-white pigmentation. Over time, the coloration transitioned to a blackish olive-green hue, often accompanied by the development of a greyish halo surrounding the colonies (Figure 2). Microscopic examination, conducted on day five using lactophenol blue staining, revealed the presence of septate hyphae, indicative of the fungal nature of the isolate. These hyphae were observed to be interspersed with simple or branched conidiophores, which exhibited a distinct curved configuration at the points of origin of the conidia, creating a characteristic zigzag appearance (Figure 3). The conidia themselves displayed a thickened cell wall and an oblong-cylindrical shape with rounded ends. Additionally, the presence of transverse septa within the conidia was evident. The distinct morphology of these structures is crucial for the preliminary identification of the pathogenic fungal species. However, due to the morphological similarities with other species within the *Curvularia* genus, molecular testing was essential for precise identification. To achieve this, genomic DNA was extracted from the cultured isolate using the EZ1 DSP DNA Blood Kit (Qiagen, Heidelberg, Germany). The DNA was then amplified using the universal fungal-specific primers ITS3F (5′-GCATCGATGAAGAACGCAGC-3′) and ITS4R (5′-TCCTCCGCTTATTGATATGC-3′), which target the internal transcribed spacer (ITS) regions of the fungal ribosomal DNA, as described by White et al. (1990) [6]. Polymerase chain reaction (PCR) amplification was performed using Platinum™ SuperFi™ DNA Polymerase (Invitrogen™, Thermo Fisher Scientific, Waltham, MA, USA), in a GeneAMP PCR System 9700 (Applied Biosystems, Foster City, CA, USA). The thermal cycling conditions were as follows: an initial denaturation at 98 °C for 30 s, followed by 35 cycles of 98 °C for 10 s, 55 °C for 10 s, and 72 °C for 1 min and 10 s. A final extension was carried out at 72 °C for 5 min. PCR products were visualized on a 3% agarose gel, stained with ethidium bromide, to confirm successful amplification. Amplicons were then purified using the Illustra™ ExoProStarTM kit (GE Healthcare, Cytiva, Marlborough, MA, USA) and sequenced with the BigDye Terminator Cycle Sequencing Kit (Applied Biosystems, Foster City, CA, USA) on a 3500xL Dx Genetic Analyzer sequencer (Applied Biosystems, Foster City, CA, USA). Nucleotide sequences were analyzed using Sequencher™ software 4.1.4 (Gene Codes Corporation). The resulting consensus sequence was submitted to the GenBank BLAST database, version BLAST 2.16.0 (URL: https://blast.ncbi.nlm.nih.gov/Blast.cgi, accessed on 28 October 2024 and 19 November 2024) for further comparison. The sequence analysis revealed that both clinical isolates showed a query cover of 99% with *Curvularia spicifera* (accession numbers MW063635.1 and PQ203619.1), confirming the species identification. This molecular characterization provided confirmatory species-level identification, supporting the morphological findings, particularly in the context of potential overlap among dematiaceous fungi.

## 4. Discussion

The two cases presented describe *Curvularia spicifera* as an etiologic agent of non-invasive fungal rhinosinusitis in immunocompetent individuals. Although *Aspergillus* species remain the most frequently implicated pathogens, dematiaceous fungi have also been reported as significant contributors, particularly in patients with type-2 inflammatory disorders [7] such as chronic rhinosinusitis with nasal polyposis. To better contextualize our findings, we performed a literature review of previously reported cases of AFRS caused by *Bipolaris spicifera* (currently classified as *Curvularia spicifera*) in immunocompetent patients (Table 1). The clinical manifestations, such as nasal obstruction, rhinorrhea, mucostasis, and hyposmia, are very unspecific, making it difficult to associate them with a specific pathology. In our cases, radiologic evidence of hyperdense material suggested fungal concretions, while histopathology confirmed prominent eosinophilic inflammation, consistent with an allergic fungal disease. The identification of *C. spicifera* was made through the culture of surgically drained mucin, underscoring the role of microbiological examination as the gold standard for diagnosis, particularly given the lack of serological assays for this species. Although culture remains a cornerstone of diagnosis, it has intrinsic limitations, including variable sensitivity and the inability to reliably distinguish colonization and contamination from pathogenic involvement. In the context of AFRS, where fungi are embedded within allergic mucin, culture results must always be interpreted alongside clinical, histopathological and radiological findings. Notably, most cases identified in the literature show a comparable clinical presentation and similarly rely on culture-based identification [4,5,8,9,10,11,12,13] (Table 1). For the diagnosis of allergic fungal rhinosinusitis not caused by *Curvularia spicifera*, the assessment of total serum IgE levels may prove to be a useful diagnostic tool. This test is highly specific for fungal species such as *Penicillium notatum*, *Helminthosporium halodes*, *Mucor racemosus*, and *Alternaria alternata*, which are potent allergens that trigger the host’s immune response. However, it should be noted that this allergen test does not detect IgE immunoglobulins against *Curvularia spicifera* [4]. Neither of our patients exhibited systemic conditions predisposing them to invasive fungal infections, supporting the notion that dematiaceous fungi can cause significant sinonasal disease in immunocompetent hosts. From a diagnostic standpoint, both cases fulfilled key features consistent with AFRS according to the Bent and Kuhn criteria, including the presence of nasal polyposis, characteristic radiologic findings, eosinophil-rich mucin, and the demonstration of fungal elements without tissue invasion [14]. In contrast, fungus ball is typically characterized by dense fungal concretions in the absence of an eosinophilic inflammatory response and usually presents as a localized, non-polypoid disease. A limitation of the present study is the lack of complete immunological data, including total serum IgE levels and peripheral eosinophil counts, which are commonly used to further support the diagnosis of AFRS.

Both cases were managed with endoscopic sinus surgery, and moreover, these cases presented with a conspicuous component of eosinophilic infiltrates in bioptic specimens of mucosa. This histopathological condition also bears similarities to the histological features seen in allergic bronchopulmonary aspergillosis [3,4,5,15]. From an immunological perspective, chronic inflammatory disorders are strongly associated with specific HLA alleles, particularly those of the MHC class II. Recent studies have revealed significant links between class II HLA alleles and allergic fungal rhinosinusitis, as well as allergic bronchopulmonary aspergillosis. These findings suggest a genetic predisposition influenced by factors within the immune system [16]. Although some predispositions are known, postoperative management was limited to controlling the underlying inflammatory diathesis but not targeting fungal persistence. Consistent with our observations, most cases reported in the literature also emphasize surgical management combined with the control of the inflammatory component, while the role of antifungal therapy remains variable and not standardized.

## 5. Conclusions

Our cases of two young immunocompetent patients both undergoing a revision surgery perfectly represent the fact that a chronic disease may relapse. These cases underscore the importance of an integrated diagnostic approach combining clinical, radiological, histopathological, and microbiological data in the evaluation of non-invasive fungal rhinosinusitis diagnosis and to guide appropriate management. Further research is warranted to better define host susceptibility factors and to refine diagnostic criteria and management strategies for this condition. Overall, our findings, together with the limited available literature, support *Curvularia spicifera* as a reported etiological agent of non-invasive fungal rhinosinusitis in immunocompetent individuals.

## Figures and Tables

**Table 1:** This table presents the clinical characteristics, immune profile, treatment, and outcomes of cases of allergic fungal rhinosinusitis caused by *Bipolaris spicifera* (one of the outdated names more commonly used in the literature referring to *Curvularia spicifera*) in immunocompetent patients. The data include the patients’ age, clinical manifestations, method of infectious agent identification, antifungal treatment administered (if applicable), and treatment outcomes, as evidenced by improvements or recurrences.

Reference	Age	Clinical Manifestation	Infectious Agent Identified	Immunity Profile	Source of Infection	Identification Method	Antifungal Treatment	Outcome
Buzina et al., 2003 [4]	19	Restricted nasal breathing for 1 year, total nasal obstruction for more than 4 months, massive polyposis, fungus balls in sinuses	*Bipolaris spicifera*	Immunocompetent	Likely environmental exposure to *Bipolaris* spores, common in hot, dry climates (e.g., Kuwait)	Fungal cultures, morphological identification on Sabouraud agar, GMS staining, PCR and sequencing of the internal transcribed spacer region of the ribosomal gene cluster.	Systemic itraconazole (100 mg twice daily for 6 weeks, then 100 mg daily for 8 weeks), systemic steroids (betamethasone), topical steroids (budesonide), saline nasal douches	Improvement post-surgery with no further fungal invasion, observed mucosal healing after antifungal and steroid treatment.
Taguchi et al., 2004 [5]	70	Diplopia, bilateral nasal obstruction, nasal discharge	*Bipolaris spicifera*	Immunocompetent	Not specified	Squash cytology of the contents of the paranasal sinuses, microscopic examination of fungal hyphae, microbiological identification.	-	Allergic fungal sinusitis.
Taguchi et al., 2007 [8]	70	Bilateral nasal obstruction, purulent nasal discharge, double vision	*Bipolaris spicifera*	Immunocompetent	Likely environmental exposure (fungus in sinuses)	Squash cytology (Papanicolaou and Grocott stains), culture on Sheep Blood and Czapek–Dox agar.	-	Allergic fungal sinusitis.
Gourley DS et al., 1990 [9]	Patient 1: 16 Patient 2: 20 Patient 3: 40	Patient 1: pansinusitis, nasal polyps, frontal pain, nasal obstruction and bilateral tecanthus. Patient 2: pansinusitis, nasal polyps, headaches, periorbital swelling, and medial orbital wall bone erosion. Patient 3: intermittent nasal congestion and headaches, as well as recurrent nasal polyps.	*Bipolaris spicifera*	Immunocompetent patients	Aeroallergens, probably in the environment where the patients lived (Southern United States)	The identification process was conducted through fungal culture and microscopic observation of conidia and conidiophores. A histological examination was also conducted, which includes the presence of Charcot–Leyden crystals and fungal hybrids.	-	The surgical intervention involved the excision of allergic mucin, with no evidence of tissue or bone invasion. Postoperative recovery was positive in all cases.
Klapper et al., 1997 [10]	Case 1: 26	Mass in the left medial canthal area, epiphora, proptosis, reduced retropulsion	*Bipolaris spicifera*	Immunocompetent	Not specified	Culture on BHI agar with blood, penicillin, streptomycin; histology with Fontana–Masson stain.	No systemic antifungal therapy; surgery only	Complete resolution of symptoms after first surgery, recurrence after 2 months (on the contralateral side), resolved with second surgery.
D Ambrosetti et al. (2006) [11]	50	Paranasal sinus tumour-like lesion with orbital involvement, chronic nasal obstruction	*Bipolaris spicifera*	Immunocompetent	Probable fungal inhalation	Histology exam and fungal culture-positive.	No antifungal treatment after surgery	No recurrence after 2 years.
Coop CA, England RW, 2006 [12]	23	Right-sided proptosis, diplopia, nasal obstruction	*Bipolaris spicifera*, *Aspergillus fumigatus*	Immunocompetent	Chronic sinusitis with nasal polyposis	Skin testing (positive), silver stain on surgical specimen.	No specific antifungal treatment; treated with surgery and systemic corticosteroids	Improvement in proptosis and diplopia after surgery.
McGinnis et al. (1992) [13]	26	Brain abscess and allergic sinusitis	*Bipolaris spicifera*	Immunocompetent	Sinus infection (hematogenous spread)	Histological brain and sinus tissue exam with hematoxylin and eosin stain.	Amphotericin B + Ketoconazole	Survival.

## Data Availability

The consensus obtained was used for the GeBank BLAST (https://blast.ncbi.nlm.nih.gov/Blast.cgi). Our sequences showed in both clinical isolates a query cover of 99% with *Curvularia spicifera* (accession number MW063635.1 and PQ203619.1).
